# *In Situ* Enzyme Activity in the Dissolved and Particulate Fraction of the Fluid from Four Pitcher Plant Species of the Genus *Nepenthes*

**DOI:** 10.1371/journal.pone.0025144

**Published:** 2011-09-16

**Authors:** Yayoi Takeuchi, Michaela M. Salcher, Masayuki Ushio, Rie Shimizu-Inatsugi, Masaki J. Kobayashi, Bibian Diway, Christian von Mering, Jakob Pernthaler, Kentaro K. Shimizu

**Affiliations:** 1 Institute of Plant Biology, University of Zurich, Zurich, Switzerland; 2 Graduate University for Advanced Studies, School of Advanced Sciences, Hayama, Kanagawa, Japan; 3 Department of Limnology, Institute of Plant Biology, University of Zurich, Kilchberg, Switzerland; 4 Center for Ecological Research, Kyoto University, Otsu, Shiga, Japan; 5 Botanical Research Centre, Sarawak Forestry Corporation, Kuching, Malaysia; 6 Institute of Molecular Life Sciences, University of Zurich, Zurich, Switzerland; Purdue University, United States of America

## Abstract

The genus *Nepenthes*, a carnivorous plant, has a pitcher to trap insects and digest them in the contained fluid to gain nutrient. A distinctive character of the pitcher fluid is the digestive enzyme activity that may be derived from plants and dwelling microbes. However, little is known about *in situ* digestive enzymes in the fluid. Here we examined the pitcher fluid from four species of *Nepenthes*. High bacterial density was observed within the fluids, ranging from 7×10^6^ to 2.2×10^8^ cells ml^−1^. We measured the activity of three common enzymes in the fluid: acid phosphatases, β-d-glucosidases, and β-d-glucosaminidases. All the tested enzymes detected in the liquid of all the pitcher species showed activity that considerably exceeded that observed in aquatic environments such as freshwater, seawater, and sediment. Our results indicate that high enzyme activity within a pitcher could assist in the rapid decomposition of prey to maximize efficient nutrient use. In addition, we filtered the fluid to distinguish between dissolved enzyme activity and particle-bound activity. As a result, filtration treatment significantly decreased the activity in all enzymes, while pH value and *Nepenthes* species did not affect the enzyme activity. It suggested that enzymes bound to bacteria and other organic particles also would significantly contribute to the total enzyme activity of the fluid. Since organic particles are themselves usually colonized by attached and highly active bacteria, it is possible that microbe-derived enzymes also play an important role in nutrient recycling within the fluid and affect the metabolism of the *Nepenthes* pitcher plant.

## Introduction

As already described by Darwin [1], carnivorous plants possess special organs to consume insects and other small invertebrates. This carnivory reflects an adaptive trait to grow in low-nutrient habitats where there is a particular lack of nitrogen, phosphorous, and potassium [2]–[4]. Such plants compensate for the lack of these nutrients in the soil by catching prey, and then digesting and absorbing nutrients from it.

The pitcher plants develop jug-shaped leaves or leaf extensions to trap animals. Among the seven genera of pitcher plants, *Nepenthes* is the largest genus in the tropics and is mostly distributed in the Malesia region, with its highest diversity occurring in Borneo [5]. The pitchers have nectar that is attractive to ants and other arthropods [6]. Because the inner wall of the upper pitcher is waxy and slippery, insects lose their footing while foraging for nectar and slip down the steep walls, thus becoming trapped in the fluid of the lower pitcher. It is reported that more than 60% of the nitrogen of the plant body is derived from prey in *Nepenthes mirabilis* (Lour.) Druce [7], which suggests a relatively high degree of reliance on animal-derived nutrient in this genera. The efficiency of prey capture and digestion is thus crucial for plant growth and survival.

The proximate agents in digesting or decomposing the organic matter are extracellular enzymes within the pitcher fluid. These enzymes in part originate from the plant itself, where they are secreted by digestive cellular glands that are located in the surface of the lower zone within the pitcher [8], [9]. The characteristics of the enzymes derived from the *Nepenthes* species have been investigated in previous studies, and the enzymes include at least proteases [10]–[12], chitinases [12], [13], phosphatases [8], [14], and glucosidases [14].

On the other hand, digestive enzymes might also be produced by microbes such as fungi and bacteria that, for example, colonize insect carcasses. So far, such putatively microbial-derived enzymes have not been examined in the pitcher fluid of *Nepenthes*, even though it is known that bacteria may form in high abundance in this habitat [3], [15], [16]. Microbial-derived enzymes such as phosphatases, glucosidases, and aminopeptidases typically play a critical role in the processing of carbon, nitrogen, and phosphorus in a variety of ecosystems (e.g., fresh water [17]–[24], sea water [25], [26], and soil [27]–[31]), and microbes substantially contribute to ecosystem functioning, especially in terms of nutrient cycling. In addition, microbes that dwell on organic solids such as dead zooplankton, animal feces, and algal aggregates are often metabolically more active than their free-living counterparts, and the degradation activities of enzymes that are attached to bacterial cells and aggregated organic matter are often substantially higher than the respective activities of the dissolved enzymes in natural waters [32]. Likewise, it is conceivable that enzymes associated with the particulate fraction of the pitcher fluid of *Nepenthes*, which is composed of free-living microbes and complexes of bacteria and organic solids, might also substantially contribute to organic matter decomposition.

In this study, we aimed to profile the *in situ* enzyme activity and the abundances of free-living microbes within the pitcher fluid of four *Nepenthes* species. We also assessed enzyme activity before and after filtration of the fluid to establish whether the digestive enzymes were mainly dissolved, or if a substantial fraction was also associated with particles such as bacterial cells and other organic solids. We addressed the following specific questions.

1. What is the density of free-living microbes in the pitcher fluid of *Nepenthes*?
2. How prevalent is the digestive enzyme activity in the pitcher fluid?
3. What is the proportion of particle-bound enzyme activity in the fluid as compared with dissolved enzyme activity?

## Materials and Methods

### Study site and plant species

We used four *Nepenthes* species, *Nepenthes albomarginata* T. Lobb ex Lindl, *Nepenthes ampullaria* Jack, *Nepenthes bicalcarata* Hook. F., and *Nepenthes gracilis* Korth, which usually occur in lowland heath forest, in peat swamp forest, or at forest margins in Borneo [5]. The lid of the pitcher in *N. ampullaria* is held away from the pitcher mouth, thus allowing rainwater and fallen leaves to enter the pitcher; for the other three species, lids cover the pitcher mouth. The samples were collected within or around the Lambir Hills National Park (LHNP), Sarawak, Malaysia (4°2′N, 113°50′E; 150 m a.s.l.). *N. albomarginata* was collected near the summit of the Bukit Pantu hill (approximately 300 m a.s.l.). *N. ampullaria* was collected near a pond or at the edge of the LHNP in a relatively open and bushy area. *N. bicalcarata* and *N. gracilis* were also collected at the edge of the LHNP.

We also used the fluid of cultivated *N. ampullaria*, *N. bicalcarata*, and *N. gracilis* (a possible hybrid) from the greenhouse in the Botanical Garden of the University of Zurich, Zurich, Switzerland (47°21′N, 8°33′E; 440 m a.s.l.).

### Collection of pitcher fluid, pH measurement, and filtration procedure

Collection of field samples in Borneo took place four times, in Feb., Jun., Aug., and Nov. 2009, and cultivated plants in Zurich were sampled once, during Nov. 2009. The sample size and species varied each time because of the availability of plants on each date/site; in Borneo, 6 pitchers of *N. albomarginata* in Jun.; 2, 6, 5, 6 pitchers of *N. ampullaria* in Feb., Jun., Aug., Nov., respectively; 3, 5 pitchers of *N. bicalcarata* in Jun., Aug., respectively; 3, 6, 6 pitchers of *N. gracilis* in Feb., Jun., Nov., 2009, respectively; in Zurich, 6, 5, 4 pitchers of *N. ampullaria*, *N. bicalcarata*, and *N. gracilis*, respectively.

First, we carefully transferred the pitcher fluid to Falcon tubes using a 5–10 ml pipette in the field. All the pitchers of the Borneo plants contained prey, such as ants, termites, and flies, but also living mosquito larvae, and debris. The pitchers from cultivated plants also contained prey such as flies, but no living mosquito larvae. Because of logistic restraints in the field, the samples were stored in the dark at 4°C for up to three days prior to the enzyme assay.

Each sample was split into two subsamples, one of which was left unmanipulated (subsequently termed ‘untreated’) while the other one was filtered through a membrane syringe filter (pore size, 0.2 µm; subsequently termed ‘filtration’). Because such a filtration treatment should remove both large particles and free-living microbes from the fluid, we assumed that the filtered fluid contained only dissolved enzymes. By contrast, the untreated fluid contained both dissolved and particle-bound enzymes (subsequently termed ‘total enzymes’). Thus, any difference in enzyme activity between the untreated and filtered samples should be caused by particle-bound enzymes that, at least in part, also originate from microbes. In addition, in contrast to sterile fluid of unopened pitcher in *Sarracenia*, fluid in the unopened *Nepenthes* pitcher contains bacteria [15]. Thus, unopened pitchers were not treated as a negative control. We also initially attempted fluid sterilization by adding antibiotics such as kanamycin, ampicillin, and spectinomycin, in order to distinguish between the enzyme activity in the fluid with and without microbes. However, the pH of the fluid changed significantly after addition of these antibiotics, possibly because they induced microbial cell burst. We therefore used the filtration treatment exclusively to obtain sterile fluid.

The pH values of untreated and filtered fluid were measured using a pH meter (Huber & Co. AG, Switzerland), while we used pH paper (PANPEHA, pH 0–14, Schleicher & Schuell, USA, and pH 2.8–4.4, Toyo Roshi Co. Ltd., Japan) when the amount of the sample fluid was too small for the pH meter (N = 2 for *N. bicalcarata* in Zurich, and N = 4 for each of *N. gracilis* in Borneo (Nov. 2009) and Zurich). No significant difference between the two methods was found in the pH values (data not shown).

### Enzyme assay

We used three chromogenic substrates, 4-nitrophenyl phosphate, 4-nitrophenyl β-d-glucoside, and 4-nitrophenyl N-acetyl-β-d-glucosaminide, to estimate the activity of acid phosphatases (AP), β-d-glucosidases (BG), and β-d-glucosaminidases (NAG), respectively. We followed up by using a common colorimetric method for 4-nitrophenol linked substrates [33] to determine the enzyme activity in the pitcher fluid samples. All substrates were prepared in 50 mM (pH 5.0) acetate buffer, and the concentration of each was 5, 5, and 2 mM, respectively.

In Feb., Jun., and Aug. 2009, the assays were conducted for both untreated and filtered fluid in the laboratory of the LHNP field station as follows. Portions of 100, 150, and 100 µl of fluid from *N. albomarginata*, 200, 400, and 400 µl from *N. ampullaria*, 100, 400, and 400 µl from *N. bicalcarata*, and 100, 200, and 200 µl from *N. gracilis* were used for assessing the activity of AP, BG, and NAG, respectively. Each sample was added to a 1.5 ml tube, diluted, if necessary, to 400 µl with 50 mM acetate buffer, and mixed with 400 µl of each substrate. As controls, 400 µl of each substrate solution was mixed with 400 µl of 50 mM acetate buffer. Mixed samples were incubated at 25°C for two hours. Thereafter, 160 µl of 1.0 N NaOH was added to terminate the reaction. Absorbance was measured at 410 nm for AP, BG, and NAG with a Shimadzu UV-1200 spectrophotometer (Shimadzu, Kyoto, Japan). In Nov. 2009, the same treatment was carried out with fluid from cultivated plants in the laboratory at the University of Zurich. On that occasion, we reduced the total volume of measured sample to one-tenth of the volume that was required in the LHNP field station because of the availability of superior instrumentation. Absorbance was then measured using a Beckman DU800 microplate spectrometer (Beckman, Fullerton, California, USA), which has the same accuracy in terms of wavelength and photometrics as the UV-1200 according to the manufacturer's specifications.

Standard curves were determined for each set of measurements using 4-nitrophenol for AP, BG, and NAG. The activities were recalculated into mmol l^−1^ h^−1^. If the estimate of particle-bound activity as derived from the difference of untreated and filtered samples yielded a negative result, it was set to zero (i.e., no difference). If the activity measurement in a particular sample was already below the detection limit before filtration, it was excluded from the subsequent statistical analysis.

### Total bacterial abundance

Samples of pitcher fluid were fixed with formaldehyde (2% final concentration) and stored at 4°C until further processing. Five to 300 µl of formaldehyde-fixed samples were stained with DAPI (4′, 6-diamidino-2-phenylindole, 1 µg ml^−1^ sample [34], and filtered onto black polycarbonate filters (0.22 µm pore size, Osmonics) placed on cellulose nitrate support filters (0.45 µm pore size, Sartorius). Images of preparations were automatically recorded with a CCD camera (Zeiss Axiocam) mounted on an epifluorescence microscope (Zeiss AxioImager.Z1) equipped with a motorized stage, and analyzed by a custom-made automated counting routine programmed in Visual Basic for Applications [35].

### Statistical analyses

**Figure 1:**
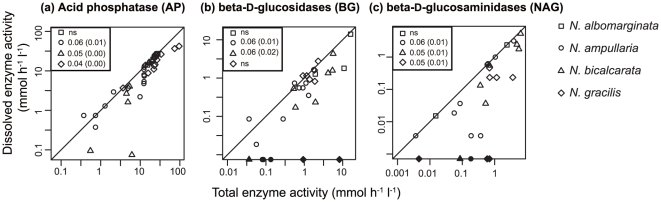
Total enzyme activity (before filtration) and dissolved enzyme activity (after filtration) in Borneo. (a) Acid phosphatases (AP), (b) β-d-glucosidases (BG), and (c) β-d-glucosaminidases (NAG) in each species. A closed symbol represents that dissolved enzyme activity was not detectable and was treated as zero. The differences in mean values between untreated and filtered fluid were examined by Wilcoxon signed-rank test, and p-values were adjusted by the BH method for multiple comparison and were shown in the upper box for each species (unadjusted p-values are shown in brackets). ns: not significant.

**Figure 2:**
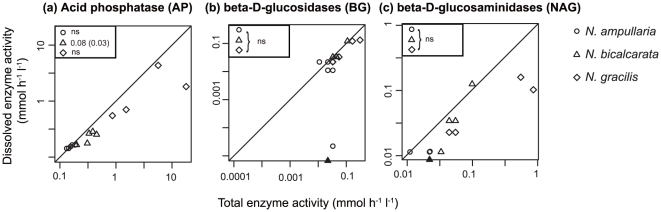
Total enzyme activity (before filtration) and dissolved enzyme activity (after filtration) in Zurich. (a) Acid phosphatases (AP), (b) β-d-glucosidases (BG), and (c) β-d-glucosaminidases (NAG) in each species. A closed symbol represents that dissolved enzyme activity was not detectable and was treated as zero. The differences in mean values between untreated and filtered fluid were examined by the Wilcoxon signed rank test, and p-values were adjusted by the BH method for multiple comparison and were shown in the upper box for each species (unadjusted p-values are shown in brackets). ns: not significant.

We examined the differences in mean pH values and enzyme activity among dates/sites within species and among species within dates/sites by applying Kruskal–Wallis tests. When the difference was significant, we also performed multiple comparisons with a *post hoc* Scheffé-type test (nonparametric). The differences in pH values and enzyme activities between untreated and filtered fluid were examined by a Wilcoxon signed-rank test for paired observations. The ratio of enzyme activity in untreated and filtered fluid was calculated and examined by a Kruskal–Wallis test. In all cases of multiple comparisons, we adjusted the p-values by a false discovery rate control method [BH method; 36]). These statistical analyses were performed using R 2.13.0 [37]. Furthermore, to explore factors that affect enzyme activity in the Borneo samples, we conducted a generalized linear mixed model (GLMM) for each enzyme using the lmer function in R (the restricted maximum likelihood (REML) approach, package lme4. Enzyme activities were log-transformed and were included as the response variable (N = 86 for AP, N = 68 for BG, N = 52 for NAG). If the activity of the filtered sample was zero, we added a small positive value, 10^−2^, because of log-transformed values (N = 7 for BG, N = 5 for NAG, i.e., closed symbols in Figures 1 and 2). We used the filtration treatment (untreated and filtration), pitcher plant species and pH (log-transformed) as explanatory variables. We included random intercepts for the pitcher ID and date that allowed intercepts to vary by them, as well as resulting in a random slope with respect to the treatment that allowed the effect to vary by pitcher ID. To evaluate the significance of parameter estimates in the fitted GLMM, we obtained a 95% confidence limit (CL) using the Markov Chain Monte Carlo (MCMC) method using the mcmcsamp function in R (package lme4). When the CL of the parameter does not include zero, this indicates that the factor differs significantly from zero at the 5% level. This multilevel modeling and MCMC procedure work reasonably well when the data contain groups with a small sample size [38].

Estimated numbers of bacterial cells per ml were log-transformed. An ANCOVA was performed to examine whether the pH and species/site were associated with the density. Posterior comparison of means after the ANCOVA used the Tukey–Kramer HSD test. ANCOVAs were implemented using the REML formula, JMP 5.0 (SAS Institute). We also tested the correlation between bacterial densities and the mean ratio of dissolved enzyme activity to total enzyme activity (filtration/untreated) by using a generalized linear model (GLM) for each enzyme.

## Results

### The characteristics of the *Nepenthes* fluid

**Figure 3:**
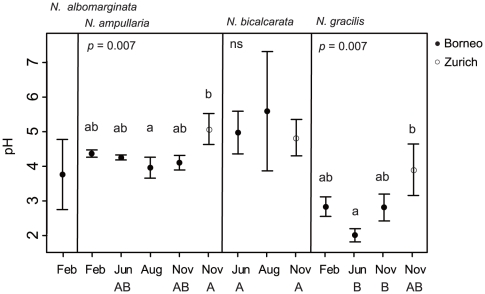
Average (SD) of pH values in each species per date/site. The differences in mean value of the pH between the dates/sites within a species were examined by the Kruskal–Wallis test (p-values adjusted by the BH method, shown above each species). Differences of a species/site between dates were also examined by the Kruskal–Wallis test (X-axis legend; Feb. and Aug.; not significant, Jun. and Nov.; p = 0.005). Results that share the same letters are not significantly different (lower case for species examined at the same date, upper case for dates between species, Scheffé-type test, p<0.05).

The pH values of *Nepenthes* fluid were acidic in all the samples (Figure 3), and did not differ for the dates of collection within the species sampled in Borneo. *N. gracilis* tended to have a lower pH compared with other species but this was not always significant through the date sequence (Figure 3). The pH did not change after the filtration treatment (Wilcoxon signed-rank test, p = 0.97).

### *In situ* enzyme activity

The averaged total enzyme activity (untreated) and dissolved enzyme activity (filtration) was calculated for the four *Nepenthes* species (Table 1, Figures 1 and 2). AP activity was always detectable in all the pitchers of all species in both Borneo and Zurich (Table 1). Likewise, BG and NAG activity was also found in all species, although there was no measurable activity of this enzyme in some pitchers of all the examined species (Table 1). Altogether, total enzyme activities did not differ significantly between the dates for either enzyme in either species (Kruskal–Wallis test, p<0.05); therefore, they were pooled for further statistical analysis.

**Table 1:** Summary of activities of the three enzymes.

					Total (Untreated)			Dissolved (Filtration)					
Site	Enzyme	Species	No. of sampled pitchers	No. of pitchers that had positive enzyme activity	Mean enzyme activity (mmol l^−1^ h^−1^)	±	SD	Mean enzyme activity (mmol l^−1^ h^−1^)	±	SD	Mean ratio of dissolved enzyme activity to total	±	SD
Borneo	Acid phosphatase	*N. albomarginata*	6	6	23.09	±	4.61	22.60	±	5.17	0.97	±	0.04
	(AP)	*N. ampullaria*	14	14	6.52	±	5.82	4.16	±	4.10	0.82	±	0.46
		*N. bicalcarata*	8	8	9.28	±	7.17	7.32	±	7.89	0.59	±	0.38
		*N. gracilis*	15	15	28.88	±	24.47	19.24	±	11.10	0.76	±	0.22
	beta-D-glucosidases	*N. albomarginata*	6	3	9.60	±	7.14	5.68	±	7.18	0.57	±	0.36
	(BG)	*N. ampullaria*	19	15	0.59	±	0.55	0.42	±	0.46	0.64	±	0.65
		*N. bicalcarata*	8	7	2.61	±	2.41	1.17	±	1.51	0.40	±	0.36
		*N. gracilis*	15	9	2.27	±	2.28	1.11	±	0.85	0.70	±	0.47
	beta-D-glucosaminidases	*N. albomarginata*	6	2	1.21	±	1.69	1.20	±	1.68	1.00	±	0.01
	(NAG)	*N. ampullaria*	19	10	0.70	±	1.16	0.55	±	0.99	0.56	±	0.45
		*N. bicalcarata*	8	7	2.64	±	2.81	1.47	±	1.95	0.41	±	0.32
		*N. gracilis*	15	7	1.07	±	1.18	0.17	±	0.18	0.18	±	0.25
Zurich	Acid phosphatase	*N. ampullaria*	6	6	0.16	±	0.02	0.16	±	0.01	0.99	±	0.06
	(AP)	*N. bicalcarata*	5	5	0.33	±	0.09	0.23	±	0.05	0.70	±	0.13
		*N. gracilis*	4	4	6.48	±	7.89	1.85	±	1.75	0.49	±	0.29
	beta-D-glucosidases	*N. ampullaria*	6	6	0.03	±	0.01	0.01	±	0.01	0.75	±	0.70
	(BG)	*N. bicalcarata*	5	5	0.05	±	0.03	0.04	±	0.05	0.72	±	0.43
		*N. gracilis*	4	4	0.14	±	0.13	0.08	±	0.06	0.61	±	0.13
	beta-D-glucosaminidases	*N. ampullaria*	6	5	0.02	±	0.00	0.01	±	0.00	0.60	±	0.22
	(NAG)	*N. bicalcarata*	5	5	0.05	±	0.03	0.04	±	0.05	0.58	±	0.46
		*N. gracilis*	4	4	0.38	±	0.40	0.07	±	0.06	0.32	±	0.17

To assess the contribution of particle-bound enzyme activity, the mean ratio of dissolved enzyme activity to total enzyme activity was calculated. The values varied from 0.2 to 1.0 in the Borneo samples and from 0.3 to 1.0 in the ones from Zurich (Table 1). Total enzyme activity was significantly lower in the Zurich samples than in the Borneo samples for all the enzymes (Mann–Whitney test, p<0.05). Similarly, the Zurich samples showed a lower dissolved enzyme activity than the Borneo samples (Mann–Whitney test, p<0.05).

### Dissolved vs. particle-bound enzyme activity

First, we tested whether the filtration reduced the enzyme activity significantly. In the Borneo samples, AP activity significantly decreased after filtration for *N. gracilis*, whereas this decrease was only marginally significant for *N. ampullaria* and *N. bicalcarata* (Wilcoxon signed-rank test, Figure 1a). BG activity decreased after filtration in *N. ampullaria* and *N. bicalcarata* (Figure 1b). For NAG, we found a marginally significant decrease in activity after filtration in *N. ampullaria*, *N. bicalcarata*, and *N. gracilis* (Figure 1c). These data support the contribution of particle-bound enzyme activities in Borneo samples. In the Zurich samples, we did not find any significantly decreased enzyme activity after filtration (Figure 2), with the possible marginal exception of AP activity in *N. bicalcarata* (Figure 2a), which might be partly because of the lower enzyme activity in the Zurich samples (also see Discussion).

Second, we tested whether the mean ratio of enzyme activity in untreated and filtered fluid differed among species. The ratios did not differ for any enzyme among the species from the Borneo samples (Kruskal–Wallis test, p>0.05 after p-value adjustment by the BH method), nor among the collection dates for the pooled species (Kruskal–Wallis test, p>0.05 after p-value adjustment by the BH method). Similarly, there were no differences among the species sampled in Zurich, except for the ratio of AP activity (Kruskal–Wallis test, p<0.02 after p-value adjustment by the BH method), which was in the order *N. gracilis*≤*N. bicalcarata*≤*N. ampullaria* (Scheffé test, p<0.05). The total enzyme activity differed among sites within the same species sampled on the same date (Nov. 2009). The activities in the Borneo samples were higher in *N. ampullaria* for AP, and in *N. gracilis* and *N. ampullaria* for BG (Kruskal–Wallis test, p<0.05 after p-value adjustment by the BH method).

To investigate the contribution of various factors including the filtration treatment, pH and species on enzyme activity, the GLMM was conducted and then confidence intervals were estimated using MCMC. The parameter estimates of the filtration treatment were negative in all three enzyme activities (−0.77 to −0.06 for AP, −7.48 to −2.13 for BG, −7.87 to −1.84 for NAG), which further supports the observation that filtration treatment decreased the activity in all enzymes (Table 2). The pH was not correlated with the enzyme activity in the three enzymes, and furthermore, the *Nepenthes* species did not have any effect to enzyme activity mostly (Table 2).

**Table 2:** Summary of the GLMM for enzyme activity.

Enzyme^a^	AIC	Random effect	Variance	Explanatory variables	Estimate	SE	*t*-value	LCL^b^	UCL^c^
Acid phosphatase	232.2	Intercept by Pitcher ID	0.50	(Intercept)	1.73	1.32	1.31	−0.37	4.00
(AP)		Slope (Filtration treatment)by Pitcher ID	0.03	Filtration treatment	−0.42	0.12	−3.50	−0.77	−0.06
		Intercept by date	0.83	Log(pH)	0.69	0.84	0.82	−0.70	2.01
				Species *N. ampullaria*	−1.41	0.44	−3.19	−2.11	−0.72
				Species *N. bicalcarata*	−0.69	0.61	−1.14	−1.73	0.21
				Species *N. gracilis*	0.83	0.61	1.35	−0.18	1.75
beta-D-glucosidases	425.7	Intercept by Pitcher ID	0.00	(Intercept)	4.58	9.46	0.49	−6.91	28.68
(BG)		Slope (Filtration treatment)by Pitcher ID	12.79	Filtration treatment	−4.82	1.11	−4.35	−7.48	−2.13
		Intercept by date	0.00	Log(pH)	1.79	6.82	0.26	−10.65	12.66
				Species *N. ampullaria*	−3.47	3.37	−1.03	−15.32	−2.24
				Species *N. bicalcarata*	−2.56	4.37	−0.59	−13.70	2.22
				Species *N. gracilis*	−1.07	3.94	−0.27	−17.59	0.06
beta-D-glucosaminidases	319.1	Intercept by Pitcher ID	0.00	(Intercept)	5.30	12.24	0.43	−16.31	30.78
(NAG)		Slope (Filtration treatment)by Pitcher ID	12.74	Filtration treatment	−4.88	1.22	−4.02	−7.87	−1.84
		Intercept by date	0.00	Log(pH)	1.08	7.84	0.14	−12.12	17.08
				Species *N. ampullaria*	−3.43	3.95	−0.87	−14.62	1.58
				Species *N. bicalcarata*	−2.09	4.25	−0.49	−14.72	2.81
				Species *N. gracilis*	−2.37	5.25	−0.45	−18.93	3.08

The standard error (SE) and *t* values of the parameters are shown.

a Enzyme activities were log-transformed.

b Lower and

c upper 95% confidence limits for each estimates (10,000 iterations).

### Bacterial abundance in the fluid

The mean bacterial density in pitchers was 7.1×10^7^ cells ml^−1^. The density in *N. ampullaria* in the Zurich samples was the lowest (7×10^6^ cells ml^−1^, Table 3), whereas that of *N. gracilis* in the Zurich samples was the highest (2.2×10^8^ cells ml^−1^, Table 3). There were no significant correlations between bacterial densities and pH (ANCOVA, F = 0.11, df = 1, p = 0.74). Bacterial densities were positively correlated with the mean ratio of dissolved enzyme activity to total enzyme activity in BG (GLM, t = 2.09, p<0.05) and NAG (GLM, t = 2.349, p<0.05), but were not correlated with that in AP (GLM, t = −1.911, p>0.05).

**Table 3:** Average concentrations of bacterial cells (×10^6^ ml^−1^).

Site	Species	No. of pitchers	Average	±	SD	
Borneo	*N. ampullaria*	6	80	±	152	B
	*N. gracilis*	6	40	±	30	AB
Zurich	*N. ampullaria*	6	7	±	4	B
	*N. bicalcarata*	5	61	±	60	AB
	*N. gracilis*	4	218	±	106	A

In the final colum, values sharing the same letters are not significantly different among species×sites (*p*<0.05, Tukey-Kramer HSD test).

## Discussion

### High *in situ* enzyme activity in the pitcher plant fluid

In this study, we profiled the *in situ* enzyme activity of the fluid in four *Nepenthes* species growing in Borneo (wild) and Zurich (cultivated). We detected activity for all of the enzymes examined in this study, AP, BG, and NAG, in the liquid of the pitchers of all four *Nepenthes* species. Compared with other environments such as freshwater, seawater, and soil (Table 4), the enzyme activity was surprisingly high in the liquid of *Nepenthes* species, up to five orders of magnitude greater than in the others. Moreover, the activity of all the studied enzymes in *Nepenthes* was much higher than that in another carnivorous plant species, *Utricularia* Table 4, [39]. Prey in the pitchers could be a most important nutrient source for plant growth and fitness in *Nepenthes*, for which it is reported that 60% of nitrogen is derived from prey [7]. A high enzyme activity in the fluid would help in the rapid decomposition of prey to maximize the efficient nutrient use within the pitcher.

**Table 4:** Comparison of enzyme activities in wide range of environments.

Enzyme		Environment	Substrate^a^	Microbial cell density (×10^5^ ml^−1^)	Microbial biomass in soil (µg g-soil^−1^) and fresh/seawater (µg l^−1^)	Activity in soils (µmol g-soil^−1^ h^−1^) and fresh/seawater (µmol h^−1^ l^−1^)	Condition	Reference
Phosphatase	Acid	Soil, Hawaii	p-NP-P	-	3620–4500	30–60	forest	28
	Acid	Soil, Kenya	p-NP-P	-	112–3151	38–97	forest, agricultural area	29
	Acid	Soil, Tahiti	p-NP-P	-	193–855	302–711	forest, agricultural area, bare	30
	Acid	Soil, USA	MUF-P	-	-	1–16	forest, agricultural area, bare	27
	Acid	Wetland soil, Great Lake	MUF-P	-	-	0.1–0.3	wetland	31
	Acid	Freshwater, Lake Kinnet	p-NP-P	-	-	3.6–10.3	particle-bound	17
	Acid	Freshwater, Lake Kinnet	p-NP-P	-	-	12.9–21.4	dissolved	17
	Acid, Alkaline	Freshwater, Great Lake	MUF-P	-	-	1.5–2.8	surface, subsurface	20
	Alkaline	Freshwater, Lake Kinnet	p-NP-P	-	-	1.8–5.8	particle-bound	17
	Alkaline	Freshwater, Lake Kinnet	p-NP-P	-	-	1.1–1.6	dissolved	17
	Alkaline	Freshwater, Lake Kuc	MUF-P	ave. 22		0.63	oligo/mesotrophic	19
	Alkaline	Freshwater, Lake Szymon	MUF-P	ave.158		0.08	hypereutrophic	19
	Alkaline	Seawater, Red Sea	p-NP-P	-	-	0.04–0.15	oligotrophic	26
	Acid	*Utricularia spp.*	MUF-P	-	-	7.6–99.9	dissolved (after filtration)	39
	Acid	*Utricularia spp.*	MUF-P	470–10200		1.8–16.0	dissolved (after filtration)	45
	Acid	*Nepenthes spp.*	p-NP-P	40–390	-	132–95957	particle-bound (untreated)	this study
	Acid	*Nepenthes spp.*	p-NP-P	-	-	74–42178	dissolved (after filtration)	this study
beta-D-glucosidases		Soil, Kenya	p-NP-BG	-	112–3151	3–20	forest, agricultural area	29
		Soil, Tahiti	p-NP-BG	-	193–855	66–194	forest, agricultural area, bare	30
		Soil, USA	MUF-BG	-	-	0.1–0.9	forest, agricultural area, bare	27
		Wetland, Great Lake	MUF-BG	-	-	0.5–1.2	wetland	31
		Fresh water, Ohio	MUF-BG	-	-	0	river	21
		Sea water, Mediterranean Sea	MUF-BG	3.1–14.7		0–0.0012	sea	25
		Fresh water, Lake Gardno	MUF-BG	-	-	0.15–0.25	surface, subsurface	20
		Fresh water, Lakes	MUF-BG	3–570	-	0.007–0.368	lakes	23
		*Utricularia spp.*	MUF-BG	-	-	1.35–2.95	dissolved (after filtration)	39
		*Nepenthes spp.*	p-NP-BG	40–390	-	11–16050	particle-bound (untreated)	this study
		*Nepenthes spp.*	p-NP-BG	-	-	0–13958	dissolved (after filtration)	this study
beta-D-glucosaminidases		Soil, Hawaii	p-NP-NAG	-	3.62–4.5	2–9	forest	28
		Soil, Kenya	p-NP-NAG	-	112–3151	2–20	forest, agricultural area	29
		Soil, USA	MUF-NAG	-	-	0.04–0.5	forest, agricultural area, bare	27
		Soil, Great Lake	MUF-NAG	-	-	0.1–0.5	wetland	31
		Soil, USA	MUF-NAG	-	-	0.01–0.76	forest, agricultural area, bare	27
		Freshwater, Lake Gardno	MUF-NAG	-	-	0.06–0.09	surface, subsurface	20
		Freshwater, Ohio	MUF-NAG	-	-	0	river	21
		Freshwater, Piburger Lake	MUF-NAG	-	1180–9260	0–0.0085	particle-bound	24
		Freshwater, Piburger Lake	MUF-NAG	-	1180–9260	0–0.008	dissolved	24
		*Utricularia spp.*	MUF-NAG	-	-	0.096–0.356	dissolved (after filtration)	39
		*Nepenthes spp.*	p-NP-NAG	40–390	-	4–23879	particle-bound (untreated)	this study
		*Nepenthes spp.*	p-NP-NAG	-	-	0–23291	dissolved (after filtration)	this study

Enzyme activity was generally higher in Borneo, which might be related to selective pressure for high enzyme production in the wild plant but also to higher activity by the indigenous microbial consortia. Moreover, the level of enzyme activity varied substantially between individual pitchers of a single species at each site and for single sampling dates. This implies that the enzyme secretion by plants and/or microbes may depend on the local condition within each pitcher; e.g., the pitcher age, the presence of plant debris, and the amount of prey could all affect the metabolic secretion of digestive enzymes. Currently, the mechanisms of accumulation or consumption of enzymes within the pitcher remain uncertain.

All investigated species exhibited high AP enzyme activity. APs are common plant enzymes of low substrate specificity that appear to be important in the production, transport, and recycling of phosphorus [40]. It has been reported that phosphorus may be the limiting nutrient in *Nepenthes* as well as in other carnivorous plants [2]. In view of such findings and our own observation of a possibly constitutive phosphatase activity in all pitchers, phosphorus may be an essential requirement in *Nepenthes*.

By contrast, we found that BG activity was not always detectable in each pitcher, especially in *N. albomarginata* and *N. gracilis*. BG is required in the degradation process of cellulose, which is a structural component of plant cell walls. Plant material such as fallen leaves could drop into the pitcher, especially with regard to *N. ampullaria* [41], which is an open-lid species, or *N. bicalcarata*, which has a large pitcher with a lid relatively distant from the pitcher mouth. For such species that are likely to receive plant material into their pitcher, BG may be essential for cellulose decomposition.

NAG is linked to the cycling of nitrogen, which is an important nutrient for *Nepenthes* [2]. Interestingly, not all examined pitchers scored positively for NAG. These enzymes are centrally involved in the breakdown of chitin, which is the hard amino sugar component of insect carapaces and microbial cell walls. The degradation of this material might also increase the ability of other enzymes to gain access to the soft body tissue within [3]. NAG should therefore be a key enzyme to obtain nitrogen and other nutrients for the plants, and it is, therefore, unexpected that some pitchers had no measurable NAG activity. Moreover, it should be noted that some of the observed NAG activity might even be related to the molting of specialized arthropods [24], as some *Nepenthes* species, for example, are known to host live mosquito larvae that complete their life cycle inside the pitchers [42].

### Bacterial abundance in the pitcher plant fluid

A high bacterial density was confirmed here even in the pitcher fluid of *N. gracilis* at an extremely low pH (Table 3), in agreement with previous findings in another *Nepenthes* species, *Nepenthes alata* Blanco [15]. The cell densities of free-living microbes were as high as, or higher than, those in productive natural aquatic environments [43]. Moreover, because we did not specifically count cells attached to larger particles, it is conceivable that the total bacterial densities in the pitchers were even higher.

### Importance of particle-bound enzyme activity

We found that the activity of all three studied enzymes decreased after filtration in various samples collected in Borneo (Tables 1 and 2, Figure 1). In addition, the ratio of dissolved to particulate BG and NAG activity increased with higher numbers of bacterial cells in the fluid. While this observation does not prove that the enzyme activity in the particulate fraction of the fluid was exclusively of microbial origin, it nevertheless suggests that both free-living and particle-bound microbes might have contributed substantially to the total enzyme activity. We also found that not all three types of enzyme activity decreased following filtration in *N. ampullaria* and *N. gracilis* (Table 1, Figures 1 and 2). Moreover, the ratio of enzyme activity between the untreated and filtered samples was highly variable throughout all of the pitchers, species, and enzymes. These data suggest that other factors related to the plant species or individual pitchers might also affect the contribution of particle-bound enzyme activity.

It has been suggested in other carnivorous plants that the microbial community would play a significant role in digestion or in the attraction of prey [3], [44]. For example, *Utricularia* species harbored plant-associated algae and bacteria in the traps; the bacterial density in the trap was also at a similar level as *Nepenthes*, e.g. 10^7^ cells ml^−1^ (Table 4, [45]). They produced significant amount of phosphatases, especially in old pitchers [45]. This might indicate that these microbes would control the nutrient flow to other components. The algae community occurring in traps of *Genlisea* species [46] also produced enzymes, but might compete for organic phosphorous with the host plant. Another example is pitchers of *Sarracenia*, which also harbored algae and bacteria producing enzymes [47]. Considered with other evidence from a nutrient tracing experiment in *Sarracenia* species [48], these microbial communities might significantly affect the nitrogen availability within the pitcher. The taxonomic composition of the bacterial community in the fluid of *Sarracenia* species has partly been investigated recently [49], [50], and it has been suggested that this is similar to an animal gut-like bacterial community. Thus, the role of the community might be related to digestion of prey, although their functions are still uncertain.

Our study also suggests, albeit indirectly, that there may be activity of enzymes derived from microbes in the investigated *Nepenthes* species. Higashi et al. [14] isolated gram positive and gram negative bacteria in the fluid of *N. hybrida*. They also suggested that these microbes would also produce digestive enzymes. Moreover, microbe-derived enzymes would have different roles on degradation of prey from plant-derived enzymes because these two kinds of enzymes had different optimum pH-ranges of enzymes. These results could indicate that, to some extent, the microbial community might play a role in the nutrient cycle and metabolism of the *Nepenthes* pitcher and act cooperatively to the host plant. To substantiate this hypothesis, further evidence is required, such as that from a proteomics study or protein sequencing, to assess the origin and relative amounts of enzymes derived from microbes. Presently, we still do not know the diversity and abundance of different specialized populations within the microbial communities in the pitcher fluids. While the high concentrations of nutrients and organic material, as well as the prey variety might foster high microbial community diversity, the plant-produced antibacterial enzymes [12] and low pH conditions might, in turn, inhibit the growth of some microbial species.

In this study, we did not focus on the effect of pitcher age and prey species on enzyme activity. In fact, it is known that fluid in the unopened *Nepenthes* pitcher contains bacteria [15]. Consistently, fluid in unopened pitchers had the same level of phosphatase activity as that in opened and matured pitchers (Takeuchi et al., data not shown, also see [14]). This might indicate that the effect of pitcher age on enzyme level would not be strong during the younger to matured stage. Because it is suggested that enzyme secretion decreased with plant age in *Utricularia* species [45], the possibility that older pitchers could also decrease enzyme secretion more or less in *Nepenthes* might not be excluded. The prey species composition and the structure of the food web in the pitcher would change in space and time as well as pitcher age [6], [15], [51]. An experimental study on these effects would be helpful for understanding plant-microbe interaction within a pitcher. In addition, future research should aim at elucidating the causes of the observed variability in particle-bound enzyme activity to provide additional evidence for the specific contribution of microbes to this activity. Eventually, a study is required of the community composition and function of the different microbial species in the pitcher fluid.
